# Annual Report of the Obstetric Practice in the Philadelphia Dispensary

**Published:** 1839-01-19

**Authors:** Joseph Warrington

**Affiliations:** Accoucheur


*Annual Report of the Obstetric Practice in the Philadelphia Dispensary.* By JOSEPH WARRINGTON, M.D., Accoucheur.

Since the Annual Report for 1837, eighty-eight women have been delivered at full time; there have also been five cases of abortion, and one of miscarriage, making ninety-three cases referrible to the Obstetric department.

In forty-eight cases of labor, of which records have been kept as fully as circumstances would admit, there were delivered twenty-eight male and twenty-one female children, one woman having twins. All the infants have done well, except one affected with cyanosis, which died in convulsions thirty-eight hours after birth. Its respiration was always feeble and its skin constantly blue.

The average duration of labour in thirty-one cases was ten and a half hours; the shortest period being one hour, and the longest seventy-two hours.

The average time for the spontaneous expulsion of the placenta in thirty-six cases was twenty-three minutes, the extremes being three minutes and three hours.

Of twenty-six cases of vertex presentation, sixteen were of the first position, eight of the second, one of the fourth, and one of the fifth.

One case occurred in which one foot and one knee presented in the first position.

In one case of impracticable labour, delivery was effected by the crotchet; and in one case of atony of the uterus, which failed to respond to the use of ergot, delivery was effected by the forceps.

One patient was attacked with metro-peritonitis, which continued ten or twelve days. Two were attacked with metritis—all recovered.

Most of the cases of midwifery which occur in the Philadelphia Dispensary are appropriated to the purposes of clinical practice.

*Twelfth month* 31*st*, 1838.

